# Gene Expression Profiling of Tricarboxylic Acid Cycle and One Carbon Metabolism Related Genes for Prognostic Risk Signature of Colon Carcinoma

**DOI:** 10.3389/fgene.2021.647152

**Published:** 2021-09-13

**Authors:** Zheying Zhang, Huifang Zhu, Qian Li, Wuji Gao, Dan Zang, Wei Su, Rui Yang, Jiateng Zhong

**Affiliations:** ^1^Department of Pathology, School of Basic Medical Sciences, Xinxiang Medical University, Xinxiang, China; ^2^Department of Pathology, The First Affiliated Hospital of Xinxiang Medical University, Xinxiang, China; ^3^Synthetic Biology Engineering Laboratory of Henan Province, School of Life Sciences and Technology, Xinxiang Medical University, Xinxiang, China

**Keywords:** TCA cycle, one carbon metabolism, colon adenocarcinoma, prognostic, signature

## Abstract

Colorectal cancer (CRC) is one of the most prevalent malignant tumors worldwide. Colon adenocarcinoma (COAD) is the most common pathological type of CRC and several biomarkers related to survival have been confirmed. Yet, the predictive effect of a single gene biomarker is not enough. The tricarboxylic acid (TCA) cycle and carbon metabolism play an important role in tumors. Thus, we aimed to identify new gene signatures from the TCA cycle and carbon metabolism to better predict the survival of COAD. This study performed mRNA expression profiling in large COAD cohorts (*n* = 417) from The Cancer Genome Atlas (TCGA) database. Univariate Cox regression and multivariate Cox regression analysis were performed, and receiver operating characteristic (ROC) curve was used to screen the variable combinations model which is most relevant to patient prognosis survival mostly. Univariable or multivariate analysis results showed that SUCLG2, SUCLG1, ACLY, SUCLG2P2, ATIC and ACO2 have associations with survival in COAD. Combined with clinical variables, we confirmed model 1 (AUC = 0.82505), most relevant to patient prognosis survival. Model 1 contains three genes: SUCLG2P2, SUCLG2 and ATIC, in which SUCLG2P2 and SUCLG2 were low-expressed in COAD, however, ATIC was highly expressed, and the expressions above are related to stages of CRC. Pearson analysis showed that SUCLG2P2, SUCLG2 and ATIC were correlated in normal COAD tissues, while only SUCLG2P2 and SUCLG2 were correlated in tumor tissues. Finally, we verified the expressions of these three genes in COAD samples. Our study revealed a possible connection between the TCA cycle and carbon metabolism and prognosis and showed a TCA cycle and carbon metabolism related gene signature which could better predict survival in COAD patients.

## Introduction

Colorectal cancer (CRC) is one of the most common malignant tumors. More than 1.2 million patients are diagnosed with colorectal cancer each year, in which over 600,000 people died, and the damage to humans is increasing. In the United States and other Western countries, the incidence of colorectal cancer is the third highest among all types of tumors, the second highest among men, and the third highest among women (Siegel et al., 2017). Although some progress has been made in the detection and treatment of colorectal cancer in recent years, the therapeutic effect has not improved, and the specific molecular mechanisms of its occurrence and metastasis are not understood. Colon adenocarcinoma (COAD) is the most common pathological type of CRC. It is therefore of great scientific importance and clinical value to reveal the pathogenesis of COAD and to find molecular markers and drug targets of the disease.

Any type of activity in life is based on a corresponding material and energy basis, the tumor is not an exception, all kinds of biological characteristics, are closely linked to metabolism (Phan et al., 2014). If we can selectively control certain metabolic pathways in tumors, it may be possible to block the corresponding malignant phenotype. The group containing one carbon atom in the organism is named one carbon unit, which includes methyl (-CH3), alkenyl (-CH2), alkynyl (-ch =), formyl (-CHO) and iminomethyl (-ch = NH) (Ducker and Rabinowitz, 2017). The metabolism related to the generation and transfer of one carbon unit is called one carbon metabolism (Newman and Maddocks, 2017). One carbon metabolism is closely related to other metabolic processes *in vivo*. One carbon unit is an important precursor of nucleic acid (pyrimidine, purine) synthesis. It participates in the methylation of DNA, RNA, protein, lipid, neurotransmitter and glutathione synthesis through adenosylmethionine (SAM), which has an important impact on the epigenetic and redox status of cells (Yang and Vousden, 2016; Zeng et al., 2019). The tricarboxylic acid (TCA) cycle is a common metabolic pathway in aerobic organisms (Anderson et al., 2018). TCA cycle is the final metabolic pathway of the three nutrients (carbohydrate, lipid and amino acid), and the hub of carbohydrate, lipid and amino acid metabolism (Cappel et al., 2019). The TCA cycle is the main path to generate energy ATP. It regulates energy production in mitochondrial respiration and plays an important role in carbohydrate metabolism (Che-Othman et al., 2020). More and more studies have shown that the key enzyme activities of one carbon metabolism and TCA cycle are related to a variety of tumors (Vander Heiden and DeBerardinis, 2017; Anderson et al., 2018; Rizzo et al., 2018; May et al., 2019; Nie et al., 2020).

In this study, we used bioinformatics analysis guided by public databases to search for differentially expressed genes of carbon metabolism and the tricarboxylic acid cycle pathway. Univariate Cox regression, multivariate Cox regression analysis and receiver operating characteristic (ROC) curve were used to screen the gene combinations which were most relevant to patient prognosis survival. And the expression of genes in COAD tissues was verified. This study may reveal new research directions and therapeutic targets and provide a basis for the diagnosis and treatment of COAD.

## Materials and Methods

### Data Mining From TCGA

The gene expression FPKM data of COAD patients were downloaded from the TCGA portal^1^, along with their clinical data such as age, sex, tumor stage, TNM classification and survival status (project ID: TCGA = COAD). A total of 524 cases were downloaded from the TCGA database. Cases with a survival time of fewer than 30 days survival status and empty value were deleted, and there are 417 cases, which contained 379 tumor samples and 38 normal samples.

### Tissue Samples

We collected 20 pairs of COAD samples and adjacent normal tissues at the First Affiliated Hospital of Xinxiang Medical College (Xinxiang, China). All cases were pathologically confirmed as adenocarcinomas. This study is approved by the Ethics Committee of Xinxiang Medical University, and all aspects of the study follow the Declaration of Helsinki. All patients provided signed informed consent forms. The COAD tissues and adjacent normal tissues were immediately frozen in liquid nitrogen for 5 min, and stored at −80°C.

### Extraction of TCA Cycle and One Carbon Metabolism Genes

From the GSEA website^2^, we downloaded the c2.cp.kegg.v7.2 symbol file for finding the TCA and one carbon metabolism pathway genes (Subramanian et al., 2005). The expression levels of TCA and one carbon metabolism pathway genes were extracted from the expression matrix. A total of 47 genes were extracted (Supplementary Data 1).

### Differential Expression Analysis

We conducted differential expression analysis using the limma packages for TCGA data by R software. Differentially expressed genes (DEGs) were screened out using the cutoffs of FDR < 0.05 and |logFC| > 0.6 for the comparison between tumor and non-tumor samples. Accordingly, DEGs of the TCA cycle and one carbon metabolism genes were identified. Then heatmap and volcano plot were drawn by R software. Perl scripts were used to merge differentially expressed genes files and survival information files.

### Searching for Prognostic Models and Construct Nomograms

We used the ROC curve to determine the accuracy of different variables in predicting patient survival. By comparing different models, we found the optimal prediction combination. And a nomogram was drawn. ROC curve and nomogram were performed using the statistical software EmpowerStats (^3^, X&Y Solutions, Inc., Boston, MA, United States) (Zhao et al., 2019). In addition, we tested the correlation of SUCLG2P2, SUCLG2 and ATIC which were screened out by area under curve (AUC) to clinical features by using EmpowerStats software with *p* < 0.05 being considered meaningfully.

### Bioinformational Analysis of SUCLG2 and ATIC Expression From Public Database

We used driverDBV3 database^4^ to detect the SUCLG2 and ATIC expression levels in different cancer types (Liu et al., 2020). GEPIA^5^ is a newly developed online web server for analyzing the RNA sequencing expression data from The Cancer Genome Atlas (TCGA) and the Genotype-Tissue Expression (GTEx) projects (Tang et al., 2017). We used GEPIA to analyze SUCLG2 and ATIC mRNA expression levels. UALCAN database^6^ used TCGA level 3 RNA-seq and clinical data from 31 cancer types (Chandrashekar et al., 2017). It can analyze the effect of gene expression levels and clinicopathologic features. UALCAN also provides a protein expression analysis using data from Clinical Proteomic Tumor Analysis Consortium (CPTAC) dataset. UALCAN was used to analyze SUCLG2 and ATIC expression levels according to cancer types or pathological stages.

### Survival Analysis of SUCLG2P2, SUCLG2, and ATIC

EmpowerStats software was used to analyze the survival of SUCLG2P2, SUCLG2 and ATIC from TCGA data downloaded. GEPIA database was also used to analyze the relationship between SUCLG2P2, SUCLG2, ATIC and survival in COAD patients. TISIDB database^7^ was used to analyze the survival of SUCLG2 and ATIC in different cancers (Ru et al., 2019).

### Analysis of SUCLG2P2, SUCLG2, and ATIC Co-expressed Genes

In this study, the GEPIA database was used to get the co-expressed genes of SUCLG2P2, SUCLG2 and ATIC in COAD. These co-expressed genes were imported into the String database to gain interactions value among co-expressed genes. Cytoscape software was used to construct co-expression networks (Shannon et al., 2003).

### Reverse Transcription-Quantitative Polymerase Chain Reaction

We extracted RNA using TRIzol reagent and RNA reverse transcription of cDNA (Takara Co., Ltd., Dalian, China) according to the manufacturer’s instruction. Reverse transcription-quantitative polymerase chain reaction (RT-qPCR) analysis was performed using SYBR Green I (Takara Co., Ltd.) in triplicate. The results were normalized to the expression of GAPDH. The primer sequences used were listed in Supplementary Table 1. GAPDH was used as an internal control.

### Statistical Analysis

Continuous variables were expressed as means with standard deviations. Categorical variables were expressed as frequencies with proportions. Student’s *t*-test and Pearson’s Chi-square test were used to determine between-group differences in means and proportions. A Cox proportional hazard model was used for univariate and multivariate analyses. All the analyses were performed using the statistical software packages R^8^ and EmpowerStats (see text footnote 3, X&Y Solutions, Inc., Boston, MA, United States). *P* < 0.05 was considered statistically significant.

## Results

### Screening and Identification of DEGs in the TCA and One Carbon Metabolism Pathway From TCGA Datasets

**FIGURE 1:**
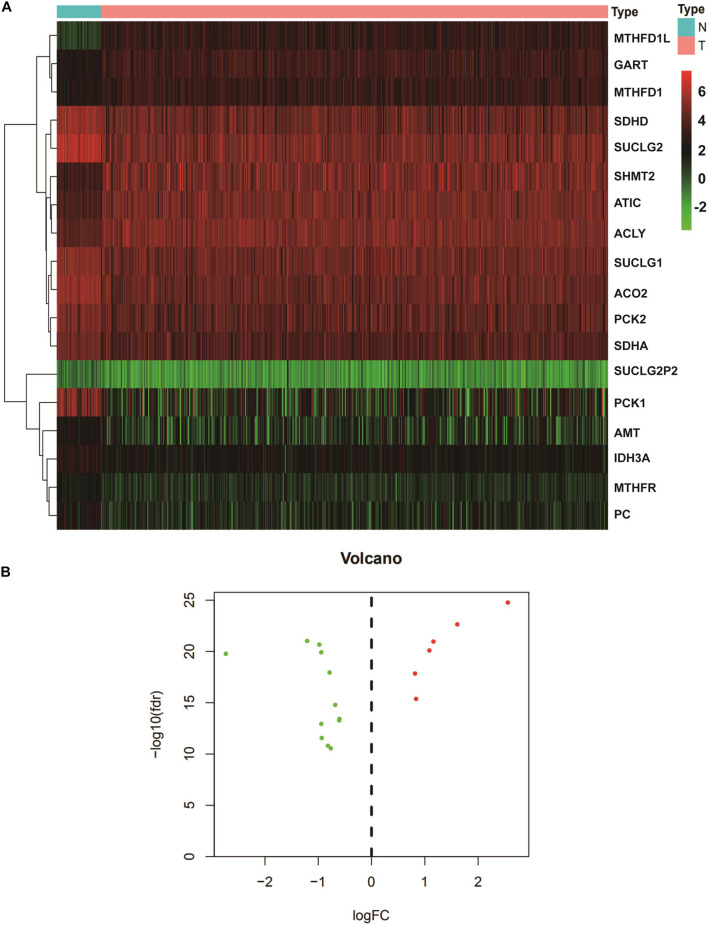
Identification of DEGs in the TCA and one carbon metabolism pathway from TCGA datasets. **(A)** The heatmap of DEGs in COAD. From red to green, the expression of genes decreased gradually. **(B)** Volcano plot of DEGs. The green represented the down-regulated genes, red represented up-regulated genes. N, normal; T, tumor. DEGs, Differentially expressed genes; COAD, Colon adenocarcinoma.

We extracted the expression levels of TCA and one carbon metabolism pathway genes from the expression matrix. We analyzed the differences between the normal group and the tumor group using the cutoffs of FDR < 0.05 and |logFC| > 0.6. A total of 18 different genes were screened out (Table 1). Then heatmap and volcano plot showed that there are six up-regulated and 12 down-regulated genes (Figures 1A,B).

**TABLE 1:** Eighteen DEGs in the TCA and one carbon metabolism pathway from TCGA datasets between tumor and normal tissues.

**Gene**	**Mean (Normal)**	**Mean (Tumor)**	**logFC**	***p* value**	**FDR**
GART	6.860483452	14.56109999	1.085737194	2.65E-21	7.96E-21
AMT	5.904272412	3.349140579	–0.817968352	1.48E-11	1.56E-11
MTHFD1L	1.891974152	11.13803961	2.557531044	9.53E-27	1.72E-25
MTHFD1	6.82431179	12.18198542	0.835993815	2.59E-16	4.24E-16
ATIC	17.34333583	38.80232075	1.161761528	2.40E-22	1.08E-21
MTHFR	3.992892931	2.621546607	–0.607016192	4.07E-14	5.24E-14
SHMT2	13.78407447	42.04375241	1.608889035	2.54E-24	2.29E-23
PCK2	44.06169612	25.9590837	–0.763285571	2.80E-11	2.80E-11
PCK1	46.12865666	6.938518342	–2.732963753	7.45E-21	1.68E-20
SUCLG2P2	0.769679814	0.402619774	–0.934840382	2.43E-12	2.74E-12
SDHD	60.72723042	31.57605843	–0.94351248	4.73E-21	1.22E-20
IDH3A	8.615992543	4.991538435	–0.787532466	5.71E-19	1.14E-18
ACLY	24.18069904	42.56273407	0.815734875	7.83E-19	1.41E-18
PC	6.165463226	3.208032005	–0.942520757	9.54E-14	1.14E-13
SDHA	33.10130754	20.63886783	–0.681524374	1.08E-15	1.62E-15
SUCLG1	50.51287079	33.28973957	–0.601573452	2.73E-14	3.77E-14
SUCLG2	92.64675618	40.10498403	–1.207958926	1.56E-22	9.39E-22
ACO2	59.76113552	30.31479509	–0.97918549	5.93E-22	2.14E-21

### Characteristics of the Study Participants

A total of 417 samples were analyzed in this study, of which 379 were tumor samples and 38 were normal (Supplementary Data 2). Table 2 shows the main baseline characteristics of the study participants. Variables including age, gender, tumor stage, TNM classification and differential genes were screened out. The variable tumor type was analyzed as column stratification. The results showed that the three variables SUCLG2P2, ACO2 genes and clinicopathological parameter stage were different between the normal group and the tumor group.

**TABLE 2:** The characteristics of patients in the TCGA-COAD cohort.

**Variables**	**Normal**	**Tumor**	***P*-value**
	*N* = 38	*N* = 379	
Age	69.4 ± 12.9	66.0 ± 12.6	0.114
Gender			0.410
Male	20 (52.6%)	173 (45.6%)	
Female	18 (47.4%)	206 (54.4%)	
Stage			0.048
I	4 (10.5%)	67 (17.7%)	
II	21 (55.3%)	140 (36.9%)	
III	5 (13.2%)	112 (29.6%)	
IV	8 (21.1%)	60 (15.8%)	
T			0.582
1	0 (0.0%)	9 (2.4%)	
2	5 (13.2%)	69 (18.2%)	
3	27 (71.1%)	257 (67.8%)	
4	6 (15.8%)	44 (11.6%)	
N			0.170
0	3 (7.9%)	62 (16.4%)	
1	35 (92.1%)	317 (83.6%)	
M			0.090
0	24 (63.2%)	287 (75.7%)	
1	14 (36.8%)	92 (24.3%)	
Gene			
GART	13.8 ± 5.6	14.8 ± 4.6	0.200
AMT	3.6 ± 3.0	3.4 ± 2.9	0.642
MTHFD1L	10.8 ± 3.7	11.1 ± 4.1	0.624
MTHFD1	12.2 ± 4.5	12.4 ± 4.2	0.822
ATIC	36.3 ± 11.3	39.8 ± 11.7	0.082
MTHFR	2.5 ± 0.8	2.6 ± 1.2	0.535
SHMT2	40.6 ± 13.5	41.4 ± 20.1	0.815
PCK2	27.7 ± 11.2	26.0 ± 11.4	0.382
PCK1	5.6 ± 4.9	7.1 ± 10.2	0.385
SUCLG2P2	0.5 ± 0.5	0.4 ± 0.3	0.041
SDHD	29.8 ± 13.6	32.4 ± 13.1	0.242
IDH3A	5.5 ± 1.9	5.0 ± 1.7	0.102
ACLY	43.5 ± 12.5	42.6 ± 12.9	0.678
PC	3.6 ± 1.8	3.2 ± 1.8	0.272
SDHA	21.4 ± 9.1	20.4 ± 7.4	0.405
SUCLG1	32.6 ± 9.8	34.2 ± 13.1	0.463
SUCLG2	38.2 ± 16.6	41.3 ± 15.8	0.244
ACO2	35.6 ± 13.2	30.0 ± 10.6	0.003

### Identification and Selection of Prognostic Related Variables

In our study, clinicopathological parameter sage, tumor stage, lymphatic, hematogenous metastases and SUCLG2, SUCLG1, ACLY, SUCLG2P2, ATIC and ACO2 genes were found to be associated with survival in univariable or multivariate analysis. In all the variables above, only lymphatic metastatic was associated with survival in both univariate and multivariate analyses. Clinicopathological parameters stage and hematogenous metastases, genes SUCLG2P2 and SUCLG2 approached significance on univariate analysis but were not predictive of survival on multivariate analysis. On the contrary, clinicopathological parameters Age, Lymphatic metastases and SUCLG1, ACLY, ATIC and ACO2 genes approached significance on multivariate analysis but univariate analysis. The *P* value of the relationship between SUCLG2P2 expression and prognosis was very close to 0.05. GART, AMT, MTHFD1L, MTHFD1, MTHFR, SHMT2, PCK2, PCK1, SDHD, IDH3A, PC, SDHA were not associated with survival (Table 3).

**TABLE 3:** Univariate and Multivariable Cox hazard regression analysis for prognostic factors.

**Variables**	**Univariate**		**Multivariate**	
	**Hazard ratio (95% confidence interval)**	***P* value**	**Hazard ratio (95% confidence interval)**	***P* value**
Age	1.0 (1.0, 1.0)	0.084	1.0 (1.0, 1.0)	0.048
Gender				
Male	1	0.313	1	0.386
Female	1.3 (0.8, 2.0)		1.3 (0.8, 2.1)	
Stage				
I	1		1	
II	2.1 (0.6, 7.2)	0.22	0.2 (0.0, 1.8)	0.132
III	4.1 (1.2, 13.8)	0.021	0.4 (0.0, 3.9)	0.397
IV	12.0 (3.7, 39.1)	<0.001	0.7 (0.1, 6.3)	0.712

**T (Tumor size)**				

1	1		1	
2	0.7 (0.1, 6.4)	0.768	0.5 (0.0, 6.0)	0.575
3	2.0 (0.3, 14.2)	0.505	1.1 (0.1, 10.8)	0.91
4	6.4 (0.9, 48.0)	0.071	2.0 (0.2, 18.8)	0.557

**N (Lymph node metastasis)**				

0	1		1	
1	12.3 (1.7, 88.4)	0.013	18.4 (1.1, 298.9)	0.041

**M (Distant metastasis)**				

0	1		1	
1	3.8 (2.5, 6.0)	<0.001	1.7 (0.7, 3.8)	0.208
GART	1.0 (0.9, 1.0)	0.514	1.0 (1.0, 1.1)	0.206
AMT	1.0 (0.9, 1.1)	0.84	1.0 (0.9, 1.1)	0.819
MTHFD1L	1.0 (1.0, 1.1)	0.648	1.0 (0.9, 1.1)	0.94
MTHFD1	1.0 (0.9, 1.1)	0.98	1.1 (1.0, 1.1)	0.097
ATIC	1.0 (1.0, 1.0)	0.095	1.0 (1.0, 1.1)	0.021
MTHFR	0.9 (0.7, 1.1)	0.261	0.8 (0.5, 1.0)	0.089
SHMT2	1.0 (1.0, 1.0)	0.595	1.0 (1.0, 1.0)	0.958
PCK2	1.0 (1.0, 1.0)	0.128	1.0 (1.0, 1.0)	0.42
PCK1	1.0 (1.0, 1.0)	0.474	1.0 (1.0, 1.0)	0.326
SUCLG2P2	0.2 (0.1, 0.6)	0.004	0.3 (0.1, 1.0)	0.057
SDHD	1.0 (1.0, 1.0)	0.362	1.0 (1.0, 1.0)	0.346
IDH3A	0.9 (0.8, 1.1)	0.294	1.1 (0.9, 1.3)	0.584
ACLY	1.0 (1.0, 1.0)	0.651	1.0 (1.0, 1.0)	0.047
PC	0.9 (0.8, 1.1)	0.227	1.0 (0.8, 1.2)	0.861
SDHA	1.0 (1.0, 1.0)	0.271	1.0 (1.0, 1.0)	0.823
SUCLG1	1.0 (1.0, 1.0)	0.077	1.0 (0.9, 1.0)	0.004
SUCLG2	1.0 (1.0, 1.0)	0.033	1.0 (1.0, 1.0)	0.216
ACO2	1.0 (1.0, 1.0)	0.504	1.0 (1.0, 1.1)	0.014

*T, represents the area of the primary tumor; T1, T2, T3, T4, with the increase of primary tumor volume, the larger the number, the greater the scope of tumor involvement; N, represents regional lymph node metastasis; N0, no lymph node metastasis; N1, lymph node metastasis; M, represents distant metastasis or not; M0, no distant metastasis; M1:, there is distant metastasis.*

### Searching for Prognostic Models and Construct Nomogram

**FIGURE 2:**
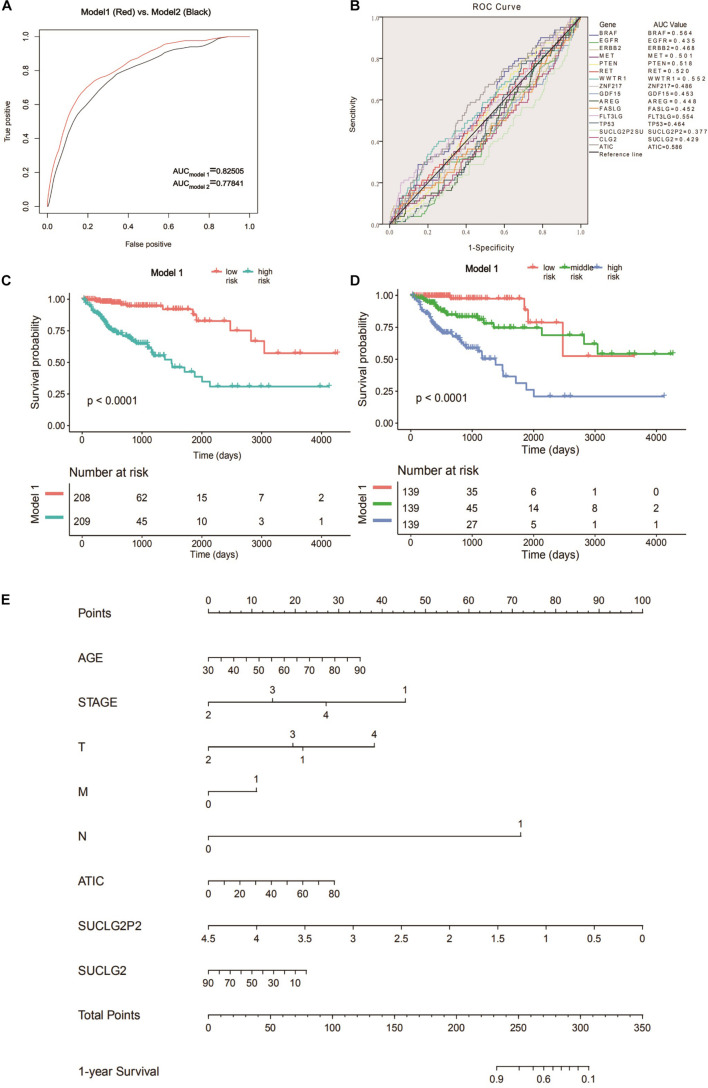
Searching for prognostic models and construct nomogram. **(A)** The ROC analysis of model 1 and model 2 for survival prediction. **(B)** ROC curves were performed for the clinically commonly used molecular markers of colon cancer and the three screened genes. **(C)** Survival curve of low- and high-risk groups stratified by model 1. **(D)** Survival curve of low- middle -risk and high-risk groups stratified by model 1. **(E)** Nomogram for predicting 1-year survival for COAD patients based on model 1. ROC, receiver operating characteristic.

Receiver operating characteristic curve is a curve drawn according to a series of different dichotomies, taking true positive rate (sensitivity) as the ordinate and false positive rate (specificity) as the abscissa, which can reflect the relationship between sensitivity and specificity. We used the ROC curve to determine the accuracy of different variables in predicting patient survival. By comparing different models, we found the optimal prediction combination (Model 1: 0.02421^∗^AGE − 1.88612^∗^(STAGE = 2) − 1.27190^∗^(STAGE = 3) − 0.75931^∗^(STAGE = 4) − 0.90448^∗^(*T* = 2) − 0.09615^∗^(*T* = 3) + 0.68434^∗^(*T* = 4) + 0.45887^∗^(*M* = 1) + 2.99163^∗^(*N* = 1) + 0.015 06^∗^ATIC − 0.92401^∗^SUCLG2P2 − 0.01040^∗^SUCLG2). The AUC is 0.82505. Compared with model 2 which is composed of a single clinical variable (AUC = 0.77841), the AUC value increased significantly (Figure 2A). ROC curves were performed for the molecular markers of colon cancer in clinical use and SUCLG2P2, SUCLG2 and ATIC genes, and we found that the AUC values were significantly lower than those of model 1 (Figure 2B). We create a new variable based on the formula in Model 1, the new variable model 1 is bisected and trisected. The Kaplan–Meier curve and log-rank test further indicated a significant difference in survival time among the third group (*P* < 0.0001) (Figures 2C,D). The variables in model 1 were used as the basis of the nomogram (Figure 2E). We also analyzed the relationship between the three selected genes (SUCLG2P2, SUCLG2 and ATIC) and clinical data. The results showed that SUCLG2P2 had an obvious correlation with clinicopathological parameters stage and distant metastasis, while SUCLG2 and ATIC had no obvious correlation with clinical data (Table 4).

**TABLE 4:** Association between SUCLG2P2, SUCLG2 and ATIC genes and clinicopathological parameters of colon cancer patients.

**Variables**	***N***	**SUCLG2P2**		***P***	**SUCLG2**		***P***	**ATIC**		**P**
		**Low**	**High**		**Low**	**High**		**Low**	**High**	
**Age**										
<60	118	58	60	0.852	59	59	0.976	59	59	0.976
≥60	299	150	149		149	150		149	150	

**Gender**										

Male	193	99	94	0.592	97	96	0.886	96	97	0.958
Female	224	109	115		111	113		112	112	

**Stage**										

I	71	30	41	0.004	33	38	0.0532	39	32	0.070
II	161	79	82		69	92		88	73	
III	117	52	65		66	51		56	61	
IV	68	47	21		40	28		25	43	

**T (Timor size)**										

1	9	4	5	0.623	3	6	0.766	5	4	0.9294
2	74	32	42		36	38		39	35	
3	284	146	138		143	141		139	145	
4	50	26	24		26	24		25	25	

**N (Lymph node metastasis)**										

0	65	28	37	0.233	31	34	0.701	36	29	0.334
1	252	180	172		177	175		172	180	

**M (Distant metastasis)**										

0	211	143	168	0.006	152	159	0.482	161	150	0.187
1	106	65	41		56	50		47	59	

*T, represents the area of the primary tumor; T1, T2, T3, T4, with the increase of primary tumor volume, the larger the number, the greater the scope of tumor involvement; N, represents regional lymph node metastasis; N0, no lymph node metastasis; N1, lymph node metastasis; M, represents distant metastasis or not; M0, no distant metastasis; M1, there is distant metastasis.*

### Bioinformational Analysis of SUCLG2P2, SUCLG2 and ATIC Expression From Public Database (driverDBV3, TCGA, GEPIA, UACLAN)

**FIGURE 3:**
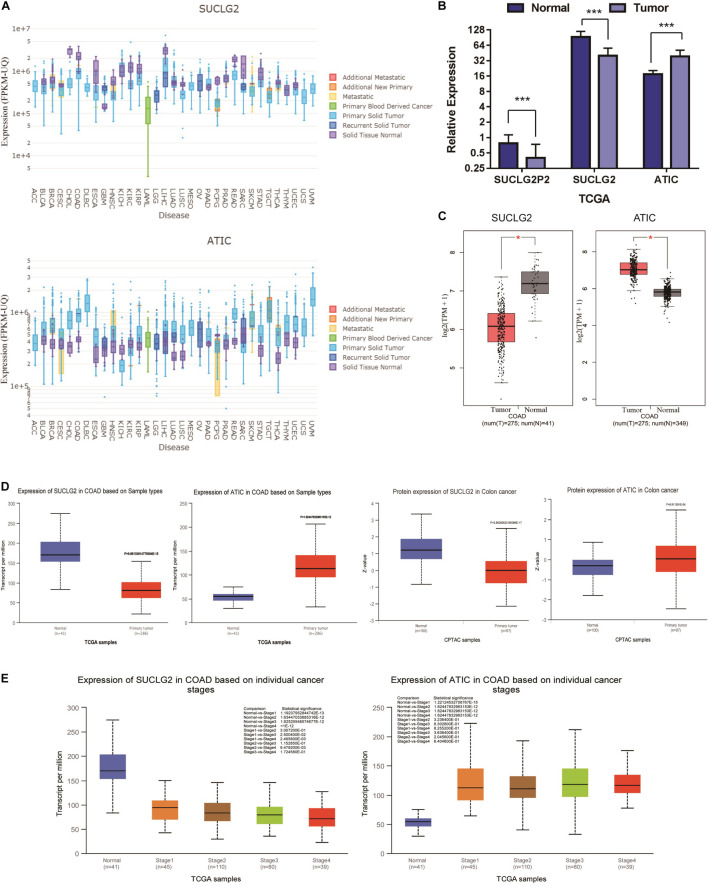
Bioinformational analysis of SUCLG2P2, SUCLG2 and ATIC expression from public Database. **(A)** The mRNA expression of SUCLG2 and ATIC in pan-cancer. **(B)** The mRNA expression of SUCLG2P2, SUCLG2 and ATIC in COAD, data is downloaded from TCGA. **(C)** The mRNA expression of SUCLG2 and ATIC in COAD from GEPIA database. **(D)** The mRNA and protein expression of SUCLG2 and ATIC in COAD from UACLAN database. **(E)** The mRNA expression of SUCLG2 and ATIC in different stage of COAD from UACLAN database. ***P < 0.001.

To determine differences of SUCLG2 and ATIC expression in tumor and normal tissues, the SUCLG2 and ATIC mRNA levels in different tumors and normal tissues of many cancer types were analyzed using the driverDBV3 database. The analysis revealed that the SUCLG2 expression was lower in most tumors compared to the normal tissues, but higher in glioblastoma. The ATIC is contrary to the expression of SUCLG2, which is expressed in most tumors (Figure 3A). Furthermore, we compared the three genes in COAD. Analyzing the downloaded TCGA data showed that the expression of ATIC was up-regulated in the tumor, and that of SUCLG2P2 was consistent with that of SUCLG2, the mRNA expression of SUCLG2P2 and SUCLG2 were down-regulated in the tumor (Figure 3B). We analyzed the expression of SUCLG2 and ATIC genes in the online public database (GEPIA and UACLAN), because none of the public databases contained the SUCLG2P2 gene, only the results of SUCLG2 and ATIC were got. The results are consistent with the analysis results of downloaded TCGA data (Figures 3C,D). Moreover, the expression of SUCLG2 and ATIC was related to the clinicopathological parameter stage (Figure 3E).

### Survival Analyses of SUCLG2P2, SUCLG2, and ATIC From Public Database (TCGA, GEPIA, TISIDB)

**FIGURE 4:**
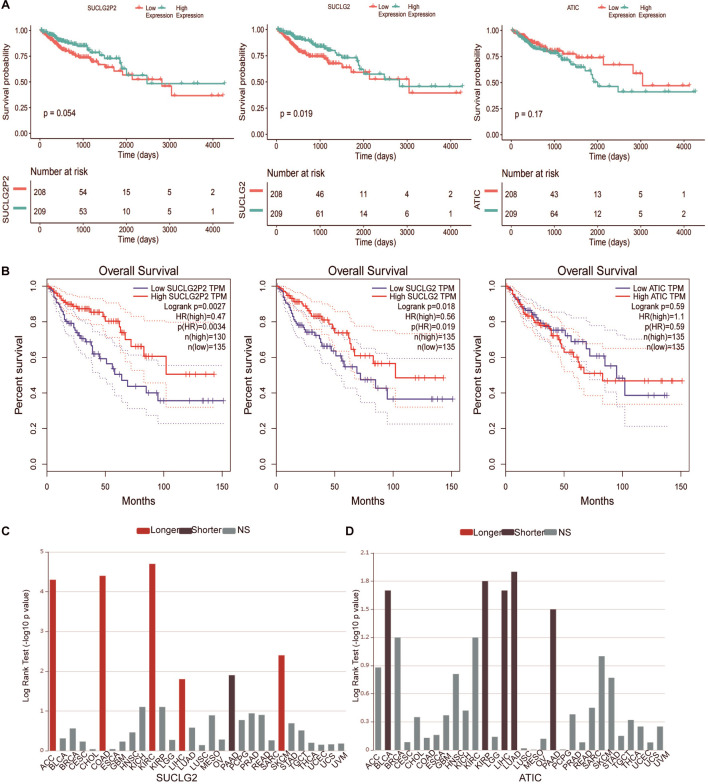
Survival analyses of SUCLG2P2, SUCLG2 and ATIC expression from public Database. **(A)** Kaplan–Meier survival curves comparing the high and low expression of three genes in COAD from TCGA downloaded data. **(B)** Kaplan–Meier survival curves from GEPIA database. **(C)** Kaplan–Meier survival curves of SUCLG2 in different types of cancer in the TISIDB database. **(D)** Kaplan–Meier survival curves of ATIC in different types of cancer in the TISIDB database. Longer represented longer survival time, Shorter represented shorter survival time, NS represented no statistical difference.

Survival analyses in the TCGA downloaded data were conducted between high and low expression groups through Kaplan–Meier analysis with the log-rank test. The results showed that SUCLG2 were closely related to survival, the *P* value of the relationship between SUCLG2P2 expression and survival was very close to 0.05 and ATIC had no obvious correlation with prognosis (Figure 4A). The results are consistent with the analysis of the online database GEPIA (Figure 4B). In the online TISIDB database, we analyzed the relationship between ATIC and SUCLG2 and prognosis in a variety of tumors (The database does not contain the data of SUCLG2P2). High expression of SUCLG2 in ACC, COAD, KIRC, LIHC and SKCM showed a higher survival rate, while low survival rate in PAAD (Figure 4C). The survival rate of ATIC with high expression in BLCA, KIRP, LUAD, LIHC and PAAD was low (Figure 4D).

### Correlation, Co-expression and Methylation Analysis of SUCLG2P2, SUCLG2, and ATIC Genes (GEPIA, STRING, MEXPRESS)

**FIGURE 5:**
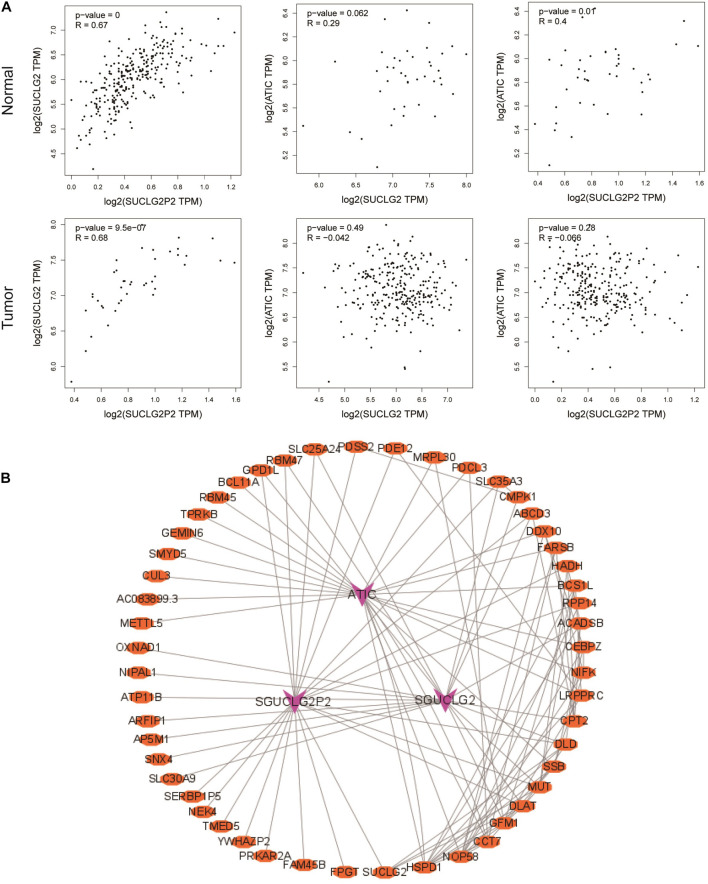
Correlation and co-expression analysis of SUCLG2P2, SUCLG2 and ATIC genes. **(A)** Pearman analysis of the correlation between SUCLG2P2, SUCLG2 and ATIC in normal tissue and tumor tissue from GEPIA database. **(B)** The co-expression network of three genes was constructed by Cytoscape software.

Correlation analysis revealed that there was a strong positive correlation among three genes in normal tissues. There was no correlation between SUCLG2P2 and ATIC, SUCLG2 and ATIC in tumor tissues (Figure 5A). Networks describing the co-expression of SUCLG2P2, SUCLG2, ATIC and similar genes can provide a deeper understanding of the associations between SUCLG2P2, SUCLG2 and ATIC and co-expression gene regulation. As shown in the network, there are interactions among the co-expressed genes of the three genes, connecting the three genes, and there may be a mutual regulatory relationship among the three genes (Figure 5B). Moreover, we take a methylation analysis to detect if the change in gene expression relating to methylation. By comparing the methylation states of the three genes, it was found that the expression of SUCLG2P2 had little relationship with methylation. SUCLG2 gene had methylation in the promoter region and also had methylation among genes, and gene expression was closely related to methylation. The methylation region of the ATIC gene was in the promoter region mainly, which was correlated with expression (Supplementary Figures 1–3). Because SUCLG2 and SUCLG2P2 have the highest correlation, and SUCLG2P2 is a pseudogene of SUCLG2 and is positively correlated. We considered the combination of SUCLG2 and SUCLG2P2 with common miRNA to achieve consistent expression and exert a tumor suppressor effect to affect the prognosis of patients. Based on the analysis results of the starBase v2.0 website, we screened out miRNA hsa-miR-588 that can bind to both SUCLG2 and SUCLG2P2 (Supplementary Figure 4).

### RT-qPCR Was Used to Verify the mRNA Expression of SUCLG2P2, SUCLG2, and ATIC in CRC Tissues

**FIGURE 6:**
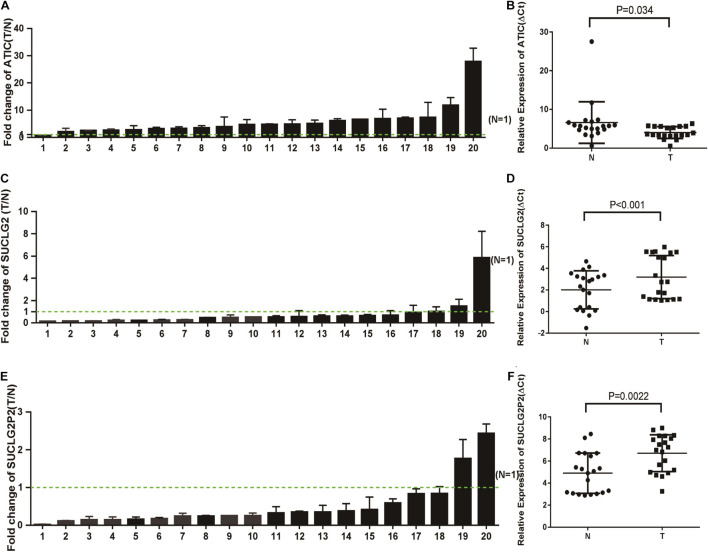
The mRNA expression of SUCLG2P2, SUCLG2 and ATIC in COAD tissues. **(A)** RT-qPCR analysis of ATIC expression in 20 paired CRC tissues (T) and adjacent normal tissues (N). ATIC expression level was normalized to GAPDH, and the results are presented as the fold change in tumor tissues relative to the matched adjacent normal tissues. Error bars indicate mean ± standard deviation (SD) of three independent experiments. Green dashed line represents T/N = 1. **(B)** The value of ΔCt was used to show the expression level of ATIC [ΔCt = Ct (ATIC) – Ct (GAPDH)] in the 20 paired human CRC tissues and adjacent normal tissues (*P* < 0.01). **(C)** RT-qPCR analysis of SUCLG2 expression in 20 paired CRC tissues (T) and adjacent normal tissues (N). **(D)** The value of ΔCt was used to show the expression level of SUCLG2 [ΔCt = Ct (SUCLG2) – Ct (GAPDH)] in the 20 paired human CRC tissues and adjacent normal tissues (*P* < 0.001). **(E)** RT-qPCR analysis of SUCLG2P2 expression in 20 paired CRC tissues (T) and adjacent normal tissues (N). **(F)** The value of ΔCt was used to show the expression level of SUCLG2P2 [ΔCt = Ct (SUCLG2P2) – Ct (GAPDH)] in the 20 paired human COAD tissues and adjacent normal tissues (*P* < 0.001).

We also confirmed the expression levels of SUCLG2P2, SUCLG2 and ATIC mRNA in tumor and non-tumor tissues from 10 patients with CRC. The RT-qPCR analysis showed significantly lower SUCLG2P2 mRNA expression in eight out of 10 CRC specimens compared with the adjacent control mucosa tissues (*P* < 0.01; Figures 6A,B). SUCLG2 mRNA expression levels in all samples were lower in tumor tissues (*P* < 0.001; Figures 6C,D). In contrast, all the mRNA expression levels of ATIC increased in tumor tissues (*P* < 0.001; Figures 6E,F). It is consistent with the results of public database analysis and proves the accuracy of the expression of the three genes.

## Discussion

Cancer metabolism has been a hot topic in cancer progression and clinical treatment. Before, it was thought that cancer cells preferred aerobic glycolysis to the TCA cycle for faster energy gain, known as the Warburg effect. In recent years, increasing studies have shown that the TCA cycle also plays an important role in tumors. Because of the Warburg effect, the energy supply of tumor cells is reduced through mitochondria. But, out of their need for rapid proliferation, tumor cells increased their demand for biosynthetic precursors and nicotinamide adenine dinucleotide phosphate (NADPH), which came from the TCA cycle. The group containing one carbon atom is named one carbon group or one carbon unit, also known as methyl groups, which comes from glycine, histidine, serine, tryptophan, methionine, etc. Metabolism related to the generation and transfer of one carbon unit is called one carbon unit metabolism. The one-carbon metabolism comprises two cycles of folic acid and methionine, thus making cells produce a carbon unit that can synthesize important anabolic precursors and methylation reactions (Ducker and Rabinowitz, 2017). One carbon unit involved the synthesis of nucleotides, S-adenosylmethionine (SAM), glutathione, and other cellular processes that are important for the rapid proliferation of tumor cells (Newman and Maddocks, 2017). By reducing folic acid in food or using anti-folic acid preparations, cells cannot get enough carbon units, the synthesis of purine, pyrimidine and other nucleotides is blocked, DNA and RNA synthesis is impaired, and the growth and proliferation of tumor cells are inhibited (Shuvalov et al., 2017; Fernandez-Villa et al., 2019). Both TCA and carbon metabolism play an important role in tumors.

We used the TCGA database to analyze genes differentially expressed in the TCA cycle and one carbon metabolism pathway in colon cancer, and then performed univariate and multivariate Cox regression analysis on these genes. Univariate analysis showed that SUCLG2P2 and SUCLG2 were correlated with survival, while no correlation was found in multivariate analysis. The possible reason is that SUCLG2P2 and SUCLG2 are correlated with survival, and there is a collinear relationship between them. Thus, in the univariate analysis, there may be significant differences between the factors SUCLG2P2 and SUCLG2, but SUCLG2P2 and SUCLG2 may not be the factors directly affecting survival. Multivariate analysis showed that SUCLG1, ACLY, ATIC and ACO2 were associated with survival, while univariate analysis showed no correlation with survival. The possible reason is that SUCLG1, ACLY, ATIC and ACO2 may be associated with other confounding factors. In univariate analysis, the actual effect of this factor is masked by the effect of other confounding factors. After eliminating the influence of other factors through multivariate analysis, SUCLG1, ACLY, ATIC and ACO2 are considered to be independent effect factors on the outcome event.

We made different combinations of the six variables of clinical data and the six screened genes to select an optimal model for prognosis evaluation (Model 1, AUC = 0.82505). Model 1 includes three genes besides clinical data. The three genes are SUCLG2P2, SUCLG2 and ATIC. Although other genes are also associated with prognosis, we finally chose SUCLG2P2, SUCLG2 and ATIC to build model 1 because we did various combinations and found that only the ROC curve where these three variables were combined had the highest AUC value. We analyzed the expressions of these three genes in different tumors. The expression of SUCLG2 was low in most tumors, especially in colon cancer. The expression of SUCLG2P2 was also low in colon cancer, but the expression of ATIC was high in most tumors, including colon cancer. We used 20 pairs of COAD tissue and adjacent tissue samples to verify the expression of three genes, which was consistent with the data analysis results. The relationship between the expression of three genes and survival was also analyzed. We found that patients with high expression of SUCLG2P2 and SUCLG2 had a longer survival time, while patients with high expression of ATIC had a shorter survival time. These results state that the three genes may play an important role in the occurrence and development of colon cancer. Through the correlation analysis between the three genes and clinical data, we found that only SUCLG2P2 was related to clinicopathological parameter stage and distant metastasis, indicating that SUCLG2P2 may have a more important function in colon cancer.

SUCLG2 is a protein coding gene that is located on chromosome 3 and has many transcripts. This gene encodes a GTP-specific beta subunit of succinyl-CoA synthetase. Succinyl-CoA synthetase catalyzes the reversible reaction involving the formation of succinyl-CoA and succinate. GTP-specific succinyl-CoA synthetase functions in the TCA, coupling the hydrolysis of succinyl-CoA to the synthesis of GTP and thus represents the only step of substrate-level phosphorylation in the TCA (Huang and Fraser, 2016). SUCLG2 plays an important role not only in neurometabolic disorders and Alzheimer’s disease (Ramirez et al., 2014; Chinopoulos et al., 2019), but also in tumors (Lin et al., 2020). It has been reported that rs35494829 of the SUCLG2 gene is associated with colon cancer (Cho et al., 2020). Besides, it has been reported that the interaction between tumor and microenvironment is inhibited by metformin through SUCLG2 (Hart et al., 2019). The results of correlation analysis between SUCLG2P2 and SUCLG2 showed that there was a significant correlation between them, and both of them were downregulated in colon cancer, which also had a high degree of homology, indicating that they may have similar mechanisms in promoting the occurrence of colon cancer, which is our further research direction. SUCLG2P2 is a pseudogene located on chromosome 12. The transcript is 1,296 bp long and has one exon, which is highly homologous with SUCLG2. There are many SNPs on the gene, and there are no other reports on the expression function of the gene. Combined with our experimental results, the low expression of SUCLG2P2 in many tumors, especially low expression in colon cancer, could be used as a diagnostic marker. SUCLG2P2 is highly homologous with SUCLG2, and is associated with colon cancer stage, metastasis and survival. It may also play an important role in the development of colon cancer. ATIC gene encodes a bifunctional protein that catalyzes the last two steps of the *de novo* purine biosynthetic pathway. It is a bifunctional enzyme that catalyzes the last two steps of purine biosynthesis (Asby et al., 2015). It is a sensitive target for chemotherapy (Liu et al., 2018). It has also been reported that it promotes the proliferation and migration of hepatocellular carcinoma by regulating the AMPK/mTOR/S6K1 signaling pathway (Li et al., 2017). Correlation analysis showed that ATIC had no correlation with SUCLG2P2 and SUCLG2 in tumor tissues, but had a correlation with normal tissues. It suggested that there may be a regulatory role between them under normal physiological conditions, yet, they are blocked in tumors. Because SUCLG2 and SUCLG2P2 have the highest correlation, and SUCLG2P2 is a pseudogene of SUCLG2, and is positively correlated. We considered the combination of SUCLG2 and SUCLG2P2 with common miRNA to achieve consistent expression and exert a tumor suppressor effect to affect the prognosis of patients. We screened out miRNA hsa-miR-588 that can bind to both SUCLG2 and SUCLG2P2. hsa-miR-588 is a tumor suppressor gene (**Qian et al., 1993**; **Yu et al., 1993**; Liu R. et al., 2020; Liu Z. et al., 2020). When the expression of SUCLG2P2 decreases in colon cancer, the combined hsa-miR-588 decreases, which increases the binding of hsa-miR-588 to SUCLG2, which in turn leads to an increase in SUCLG2 degradation and a decrease in SUCLG2 expression. In colon cancer, the expressions of SUCLG2 and SUCLG2P2 are reduced, the competitive binding hsa-miR-588 is reduced, hsa-miR-588 is increased, and hsa-miR-588 exerts a tumor suppressor effect. Thus, SUCLG2 and SUCLG2P2 may affect the prognosis of patients through hsa-miR-588. We also analyzed the relationship between the expression and methylation of the three genes. There was no significant methylation in SUCLG2P2, but there was significant methylation in both SUCLG2 and ATIC. The decreased expression of SUCLG2P2 may be regulated by other factors.

## Conclusion

In conclusion, the expressions of SUCLG2P2, SUCLG2 and ATIC in colon cancer and normal tissues were different, and they were related to survival. The model constructed by these three genes and clinical data has a good predictive ability for predicting the survival of patients. Our results suggest that SUCLG2P2, SUCLG2 and ATIC may be biomarkers or therapeutic targets for COAD. Yet, future experiments are necessary to unveil the molecular basis of SUCLG2P2, SUCLG2 and ATIC functions in COAD.

## Data Availability Statement

The original contributions presented in the study are included in the article/Supplementary Material, further inquiries can be directed to the corresponding author.

## Ethics Statement

The studies involving human participants were reviewed and approved by the Ethics Committee of Xinxiang Medical University. The patients provided their written informed consent to participate in this study.

## Author Contributions

JZ and ZZ designed the work and wrote this manuscript. HZ and WG performed the experiments. QL and DZ collected the samples. WS and RY modified the manuscript. All authors contributed to the article and approved the submitted version.

## Conflict of Interest

The authors declare that the research was conducted in the absence of any commercial or financial relationships that could be construed as a potential conflict of interest.

## Publisher’s Note

All claims expressed in this article are solely those of the authors and do not necessarily represent those of their affiliated organizations, or those of the publisher, the editors and the reviewers. Any product that may be evaluated in this article, or claim that may be made by its manufacturer, is not guaranteed or endorsed by the publisher.

## Footnotes

1
https://portal.gdc.cancer.gov/

2
http://www.gsea-msigdb.org/gsea/downloads.jsp

3
http://www.empowerstats.com

4
http://driverdb.tms.cmu.edu.tw/

5
http://gepia.cancer-pku.cn/

6
http://ualcan.path.uab.edu/

7
http://cis.hku.hk/TISIDB/

8
http://www.R-project.org

## Supplementary Material

Supplementary Figure 1 Methylation analysis of SUCLG2P2 gene from MEXPRESS database.

Supplementary Figure 2 Methylation analysis of SUCLG2 gene from MEXPRESS database.

Supplementary Figure 3 Methylation analysis of ATIC gene from MEXPRESS database.

Supplementary Figure 4 Prediction of miRNA binding sites of SUCLG2 and SUCLG2P2. **(A)** Schematic diagram of binding sites between SUCLG2 and Hsa-miRNA-588 in TargetScanHuman Database. **(B)** Schematic diagram of binding sites between SUCLG2 and Hsa-miRNA-588 in starBase v2.0 Database. **(C)** Schematic diagram of binding sites between SUCLG2P2 and Hsa-miRNA-588 in starBase v2.0 Database.
