# Supplementary figures and images for: Gene Expression Profiling of Tricarboxylic Acid Cycle and One Carbon Metabolism Related Genes for Prognostic Risk Signature of Colon Carcinoma
Source: Front Genet. 2021 Sep 13;12:647152. doi: 10.3389/fgene.2021.647152 (PMC8475515; doi:10.3389/fgene.2021.647152)

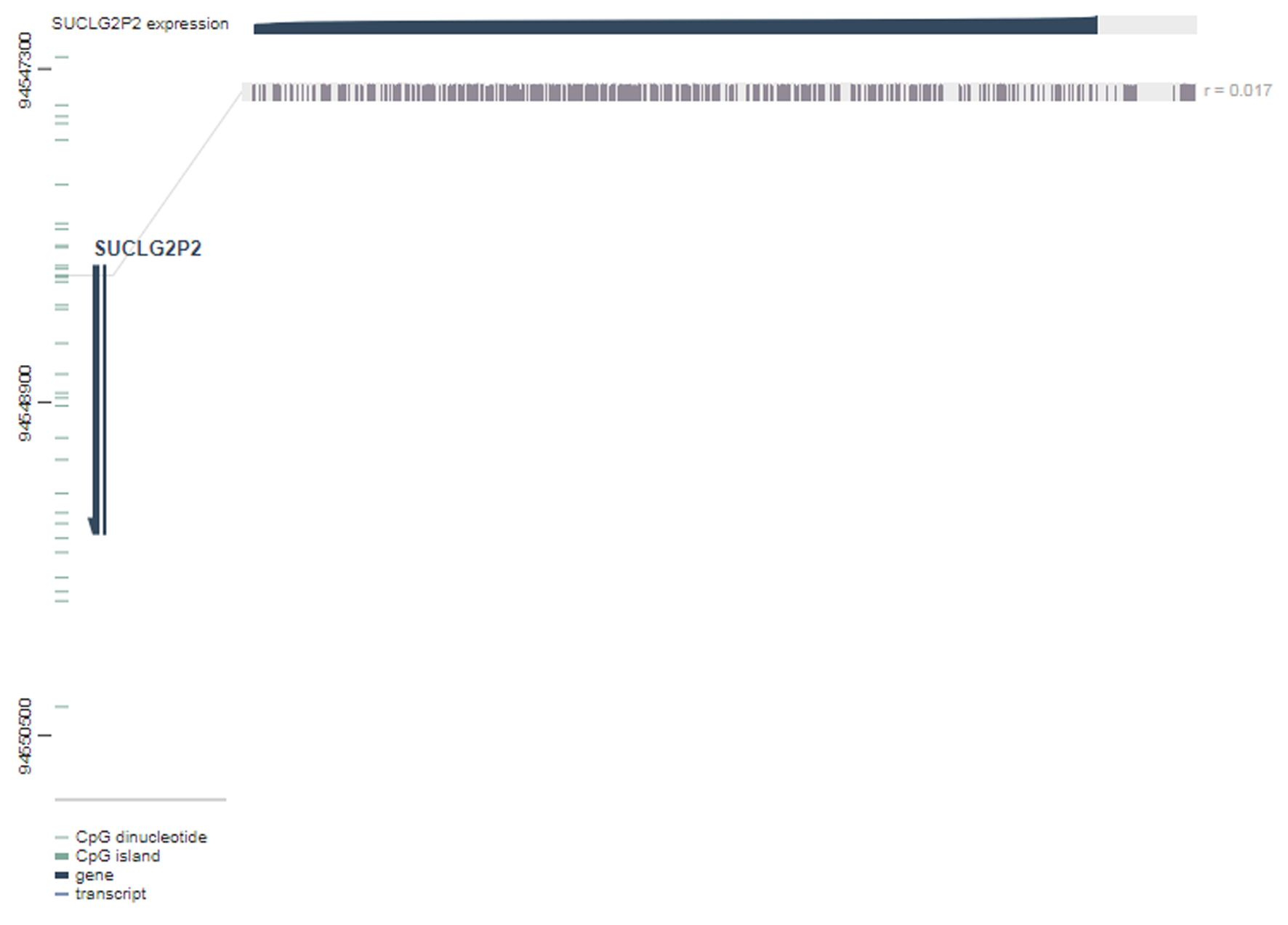

Supplement: Supplementary Figure 1 — Methylation analysis of SUCLG2P2 gene from MEXPRESS database. [file Image_1.TIF]

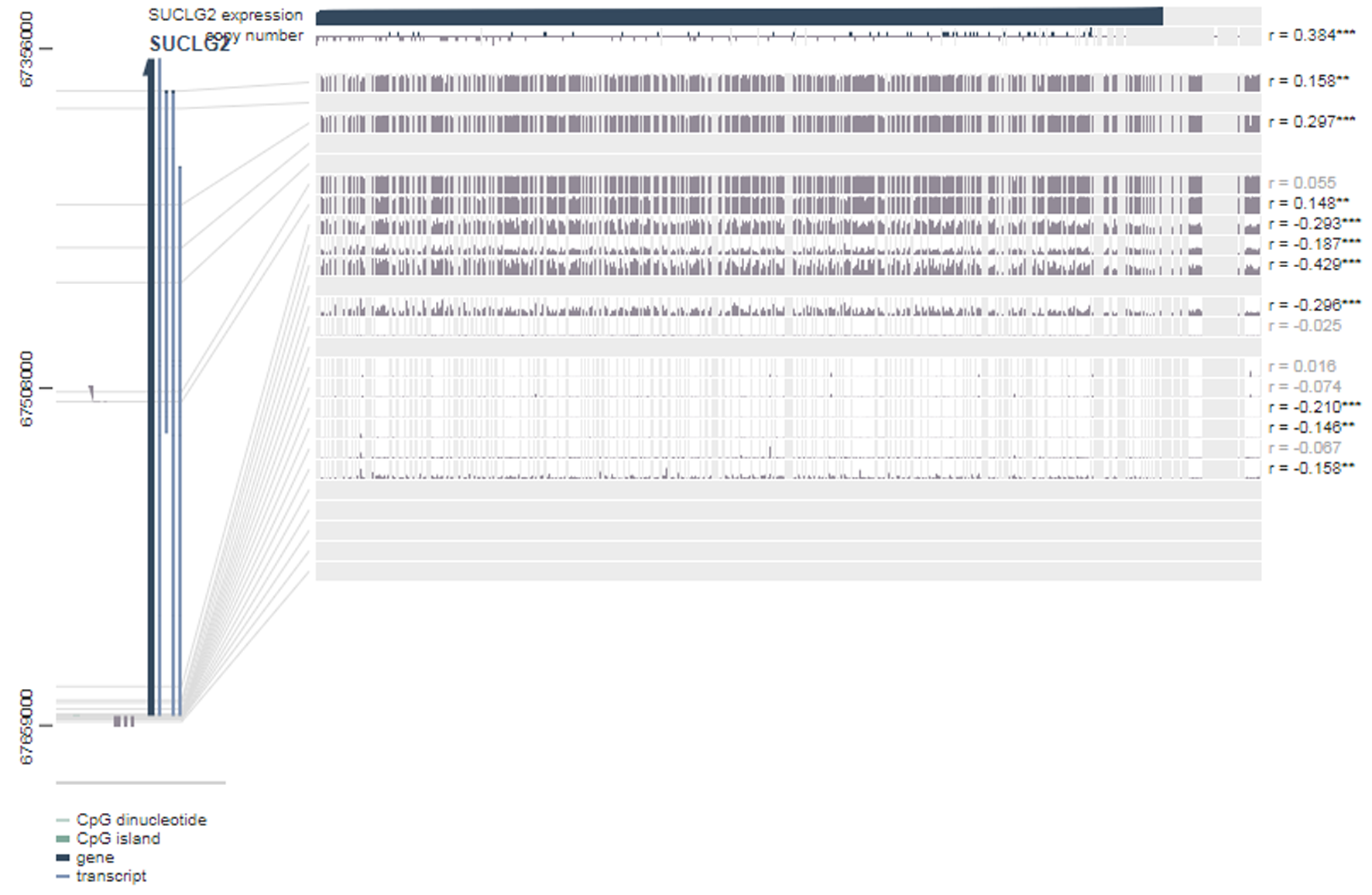

Supplement: Supplementary Figure 2 — Methylation analysis of SUCLG2 gene from MEXPRESS database. [file Image_2.TIF]

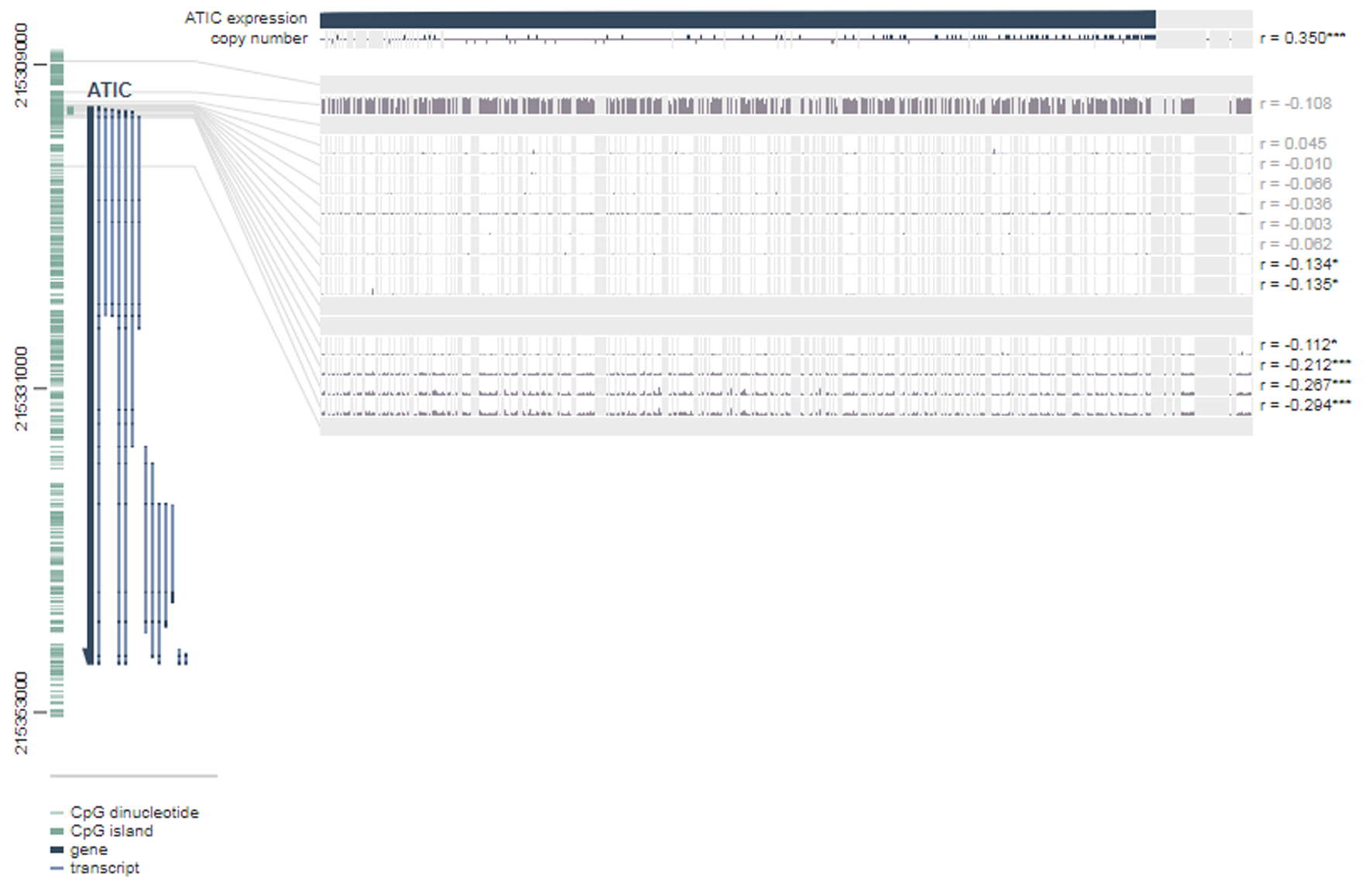

Supplement: Supplementary Figure 3 — Methylation analysis of ATIC gene from MEXPRESS database. [file Image_3.TIF]

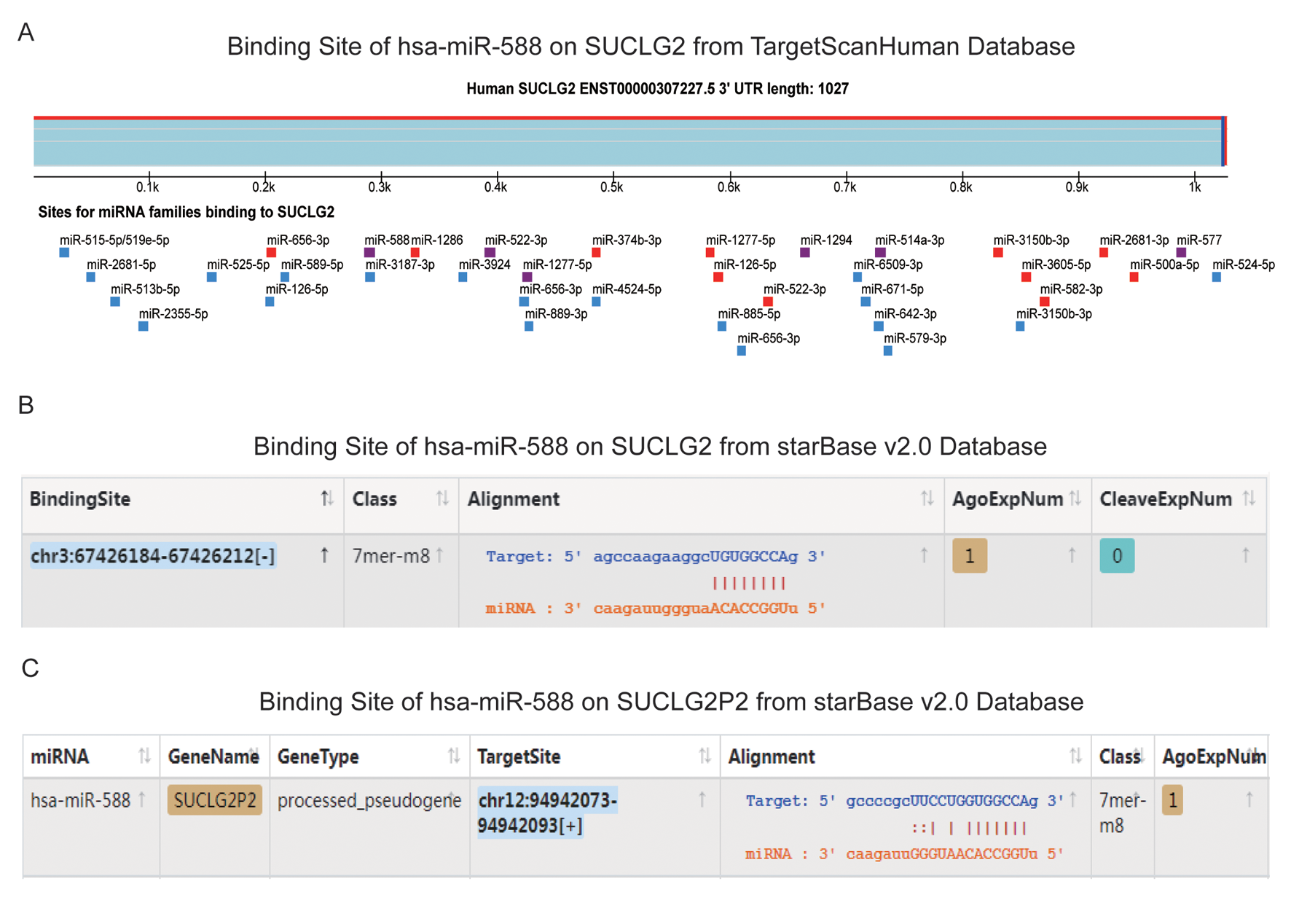

Supplement: Supplementary Figure 4 — Prediction of miRNA binding sites of SUCLG2 and SUCLG2P2. (A) Schematic diagram of binding sites between SUCLG2 and Hsa-miRNA-588 in TargetScanHuman Database. (B) Schematic diagram of binding sites between SUCLG2 and Hsa-miRNA-588 in starBase v2.0 Database. (C) Schematic diagram of binding sites between SUCLG2P2 and Hsa-miRNA-588 in starBase v2.0 Database. [file Image_4.TIF]
