# Supplementary material for: Gene Expression Profiling of Tricarboxylic Acid Cycle and One Carbon Metabolism Related Genes for Prognostic Risk Signature of Colon Carcinoma
Source: Front Genet. 2021 Sep 13;12:647152. doi: 10.3389/fgene.2021.647152 (PMC8475515; doi:10.3389/fgene.2021.647152)
Supplement: Supplementary file 5 [file Table_1.pdf]

Supplementary Table 1. Primer sequences used for qRT-PCR or PCR analyses

	Forward primer (5’-3’)	Reverse primer (5’-3’)
SUCLG2P2	GGATCTTGGAGGTGGTGT	AGGTCAACGGCTGAAGTA
SUCLG2	CAAAAGACCCTAATGTTGTGGGA	TTCAGCAACCATCACCTTGTT
ATIC	ACCTGACCGCTCTTGGTTTG	TACGAGCTAGGATTCCAGCAT
GAPDH	CTGGGCTACACTGAGCACC	AAGTGGTCGTTGAGGGCAATG
